# Optimization of the antifungal properties of the bacterial peptide EntV by variant analysis

**DOI:** 10.1128/mbio.00570-24

**Published:** 2024-04-09

**Authors:** Shantanu Guha, Shane A. Cristy, Giuseppe Buda De Cesare, Melissa R. Cruz, Michael C. Lorenz, Danielle A. Garsin

**Affiliations:** 1 Department of Microbiology and Molecular Genetics, The University of Texas Health Science Center at Houston, Houston, Texas, USA; Tel Aviv University, Tel Aviv, Israel

**Keywords:** antifungal therapy, *Candida*, *Cryptococcus*, antimicrobial peptides

## Abstract

Fungal resistance to commonly used medicines is a growing public health threat, and there is a dire need to develop new classes of antifungals. We previously described a peptide produced by *Enterococcus faecalis*, EntV, that restricts *Candida albicans* to a benign form rather than having direct fungicidal activity. Moreover, we showed that one 12-amino acid (aa) alpha helix of this peptide retained full activity, with partial activity down to the 10aa alpha helix. Using these peptides as a starting point, the current investigation sought to identify the critical features necessary for antifungal activity and to screen for new variants with enhanced activity using both biofilm and *C. elegans* infection assays. First, the short peptides were screened for residues with critical activity by generating alanine substitutions. Based on this information, we used synthetic molecular evolution (SME) to rationally vary the specific residues of the 10aa variant in combination to generate a library that was screened to identify variants with more potent antifungal activity than the parent template. Five gain-of-function peptides were identified. Additionally, chemical modifications to the peptides to increase stability, including substitutions of D-amino acids and hydrocarbon stapling, were investigated. The most promising peptides were additionally tested in mouse models of oropharyngeal and systemic candidiasis where their efficacy in preventing infection was demonstrated. The expectation is that these discoveries will contribute to the development of new therapeutics in the fight against antimicrobial resistant fungi.

**IMPORTANCE:**

Since the early 1980s, the incidence of disseminated life-threatening fungal infections has been on the rise. Worldwide, *Candida* and *Cryptococcus* species are among the most common agents causing these infections. Simultaneously, with this rise of clinical incidence, there has also been an increased prevalence of antifungal resistance, making treatment of these infections very difficult. For example, there are now strains of *Candida* auris that are resistant to all three classes of currently used antifungal drugs. In this study, we report on a strategy that allows for the development of novel antifungal agents by using synthetic molecular evolution. These discoveries demonstrate that the enhancement of antifungal activity from naturally occurring peptides is possible and can result in clinically relevant agents that have efficacy in multiple *in vivo* models as well as the potential for broad-spectrum activity.

## INTRODUCTION

*Candida albicans* is an important fungal species in the human microbiome. Its primary niche is the mammalian gastrointestinal tract and oral cavity, and it is not thought to have a major environmental reservoir (1–4). It also inhabits the urogenital tract and the skin (5–8). It is a major and frequently lethal pathogen found in immunocompromised patients, in whom it can also cause nonlethal mucosal infections, such as oropharyngeal thrush in HIV/AIDS patients (9–11). *C. albicans* is a polymorphic species, and the transition between the yeast and hyphal states is critical for virulence; the latter form is more immunostimulatory and invasive and is required for virulence and biofilm formation (12, 13). While the interaction of *C. albicans* with the host immune system has been the subject of extensive study, a developing body of literature has been uncovering specific molecular interactions between *C. albicans* and other bacterial components of the microbiome. Indeed, in previous work, we demonstrated that *Enterococcus faecalis*, a Gram-positive bacterium common in the GI tract, can impede the hyphal transition of *C. albicans* via a secreted peptide, EntV (14, 15). This peptide is a model for evolutionary adaptations of commensal species to each other and a potentially clinically relevant antifungal agent (16, 17).

Presently, there are only three broadly used classes of antifungals available, targeting ergosterol directly (polyenes), ergosterol synthesis (azoles), or the synthesis of cell wall β-glucan (echinocandins) (16, 18). There is a dire need for novel antifungal agents exemplified by the continual increase in the clinical incidence of fungal pathogens that exhibit resistance to traditional antifungal agents (19–22). Acquired antifungal resistance complicates treatment of infections from normally susceptible species like *C. albicans* and *Aspergillus fumigatus*, resulting in thousands of yearly healthcare visits and poorer outcomes for patients harboring these infections (23, 24). The highly resistant species, such as *Candida auris* and the Mucorales, present a category of infections that are essentially untreatable with current antifungals. Biofilm-based infections, such as those caused by *C. albicans* on wounds, mucosal surfaces, and medical devices, make treatment more difficult since antifungal drugs are often unable to fully penetrate the biofilms, leading to persistent and chronic infections (25, 26).

Previously, we identified a peptide bacteriocin secreted by *E. faecalis*, EntV, which exhibited strong antifungal activity against *C. albicans* (15). EntV antagonized the virulence of *C. albicans* by inhibiting biofilm formation and hyphal morphogenesis rather than acting by fungicidal or fungistatic mechanisms associated with current antifungals (15). Moreover, complete activity of EntV was shown to require posttranslational processing events including disulfide bridge oxidation by a thioredoxin, DsbA, and extracellular cleavage by the enzyme gelatinase, GelE (27, 28). Furthermore, we reported that the structure of unprocessed EntV consists of seven alpha helices, six of which form “clasping palms” enfolding the final seventh helix, α7, which becomes exposed following processing by GelE. The antifungal activity resides entirely in a 12-amino acid (aa) sequence of the α7 helix; shorter variants have less activity. Additionally, we showed that EntV and the 12aa variant were effective against drug-resistant strains of *C. albicans* and other fungal species, including *Candida auris* and *Cryptococcus* (29).

In this study, site-directed mutagenesis, select chemical modifications, as well as design and screening of a combinatorial library were used to reveal the features of α7 important for activity and whether they could be further optimized to generate shorter variants with greater antifungal activity and physiological stability. Five 10aa variant peptides were discovered that had activity comparable to that of the 12aa variant and full-length EntV in biofilm assays and the *C. elegans* infection model. Two of these library variants were further tested and were shown to have efficacy in preventing infection in vertebrate models of candidiasis—oropharyngeal and systemic. Thus, EntV-based, anti-virulence peptides hold promise for the treatment of fungal infections.

## RESULTS

### Alanine scanning and mutational analysis of EntV truncated peptides

**Fig 1:**
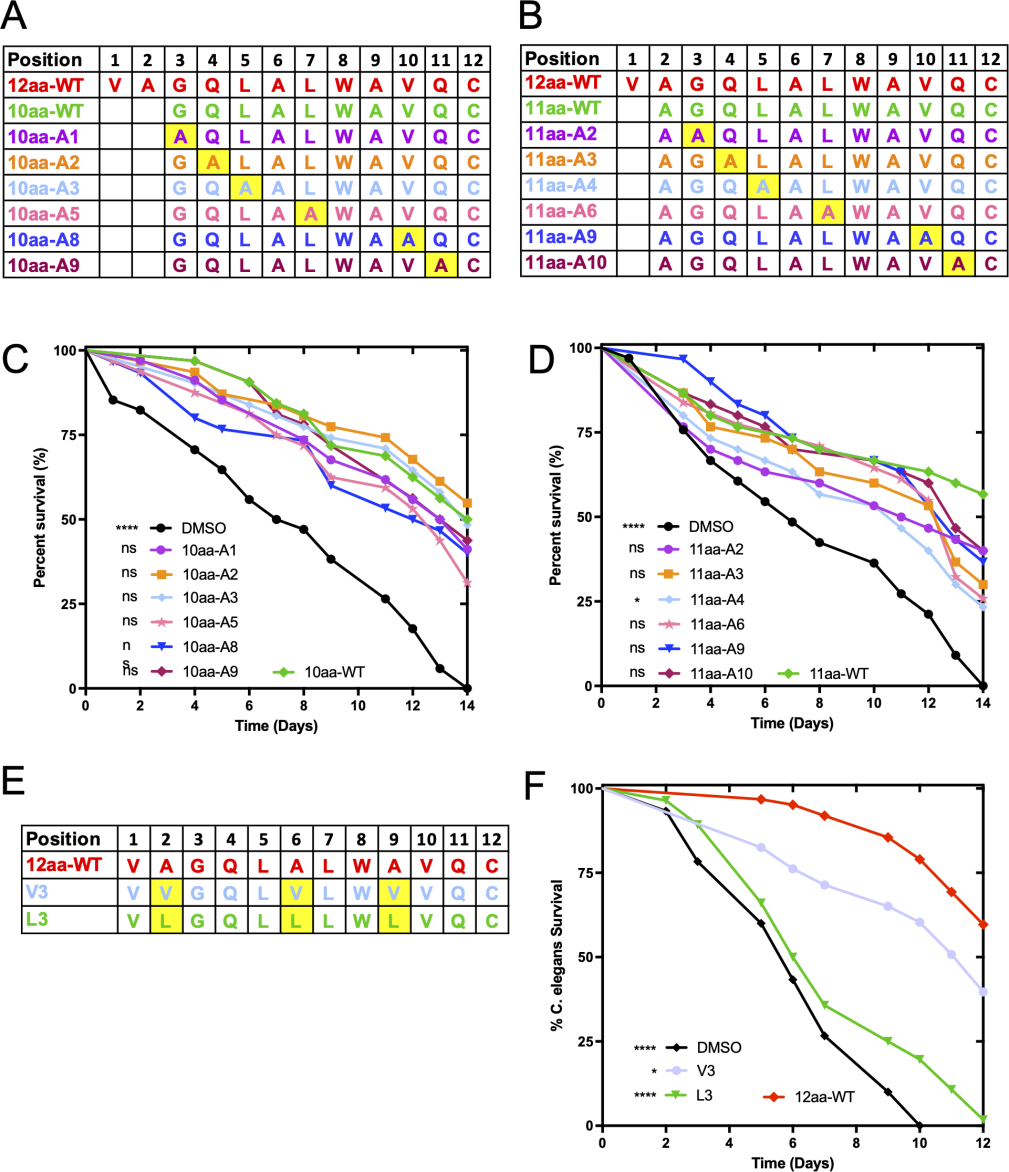
Mutational analysis of the 12aa variant of EntV. The sequences of the 10aa (**A**) and 11aa (**B**) peptides, with the alanine substitutions relative to the parental 12aa sequence. Survival over time of *C. elegans* infected with *C. albicans* and exposed to 1 nM of the 10aa (**C**) and 11aa (**D**) peptides. Survival curves were compared to those of the treatment with the WT sequence, as indicated (10aa-WT and 11aa-WT) by Mantel–Cox log rank analysis. A total of 30 animals were used, and one representative trial is shown. Median survival and *P* values of all trails are presented in Table S1. (**E**) Sequence alignments of two variants with increased overall hydrophobicity of the peptide. (**F**) Survival over time of *C. elegans* infected with *C. albicans* and exposed to 1 nM of the peptides from (**E**). The parameters and statistical analysis of this assay were the same as described for (**C** and **D**), and the replicates are shown in Table S1. For all statistical tests, *P* values < 0.05 were considered statistically significant. *P < 0.05, **P < 0.01, ***P < 0.001, and ****P < 0.0001.

Previous work showed that the cysteine in the α7 helix of EntV is important for activity. Changing it to a serine abrogated the activity in both the full-length EntV (27) and the 12aa variant (29). To identify additional key residues necessary for activity in the shorter α7 variants, alanine scanning was undertaken in which peptides containing systematic alanine substitutions were generated and tested for antifungal activity in the *C. elegans* infection model of candidiasis. The peptides generated for both the 10aa and the 11aa variants are shown in Fig. 1A and B. Residues that were not mutated included the native alanine residues found in the wild-type sequence at positions 2, 6, and 9 and the tryptophan found at position 8, which is critical for determining the peptide concentration. We had also previously determined that the two glutamines at positions 4 and 11 were dispensable for antifungal activity (29). The ability of these peptides to protect *C. elegans* from *C. albicans* infection is shown in comparison to the wild-type sequences for the 10aa and 11aa variants, respectively, in Fig. 1C and D; Table S1. Of the 12 peptide variants tested, only one, in which the leucine had been changed to alanine at position 4, 11aa-A4, had a statistically significant decrease in protection relative to the native sequence (*P* = 0.0176). Apart from those of the previously studied cysteine (27, 29), these results suggest that no other individual side-chains of the amino acids that comprise the EntV truncated peptides are critical for activity.

The α7 peptide is very hydrophobic, with over 50% of its residues being nonpolar, and we wondered if its hydrophobic nature contributes to its activity. In the previous work, we showed that replacing the two glutamines at positions 4 and 11 with isoleucine did not affect the function, but changing them to glutamic acid, which is polar and negatively charged, destroyed the activity, suggesting that hydrophobicity is preferred (29). To test this further, we assessed whether increasing the overall hydrophobicity of the peptide by incorporating valine and leucine residues in place of the native alanine residues in the wild-type sequence might be a rational way to increase the antifungal activity. Therefore, peptides were generated by replacing the three native alanine residues at positions 2, 6, and 9 to valine or to leucine and tested for antifungal activity in the *C. elegans* infection model (Fig. 1E). As shown in Fig. 1F; Table S1, the V3 variant peptide retained only partial activity, whereas the L3 peptide lost all activity. These results suggest that simply increasing the hydrophobicity of the peptide does not increase the activity and that there is some sequence specificity, with the caveat that smaller changes might have been more effective. In total, the results suggest that other than cysteine, no other residues in the α7 helix are absolutely required for activity.

### High-throughput screening of a combinatorial peptide library

**Fig 2:**
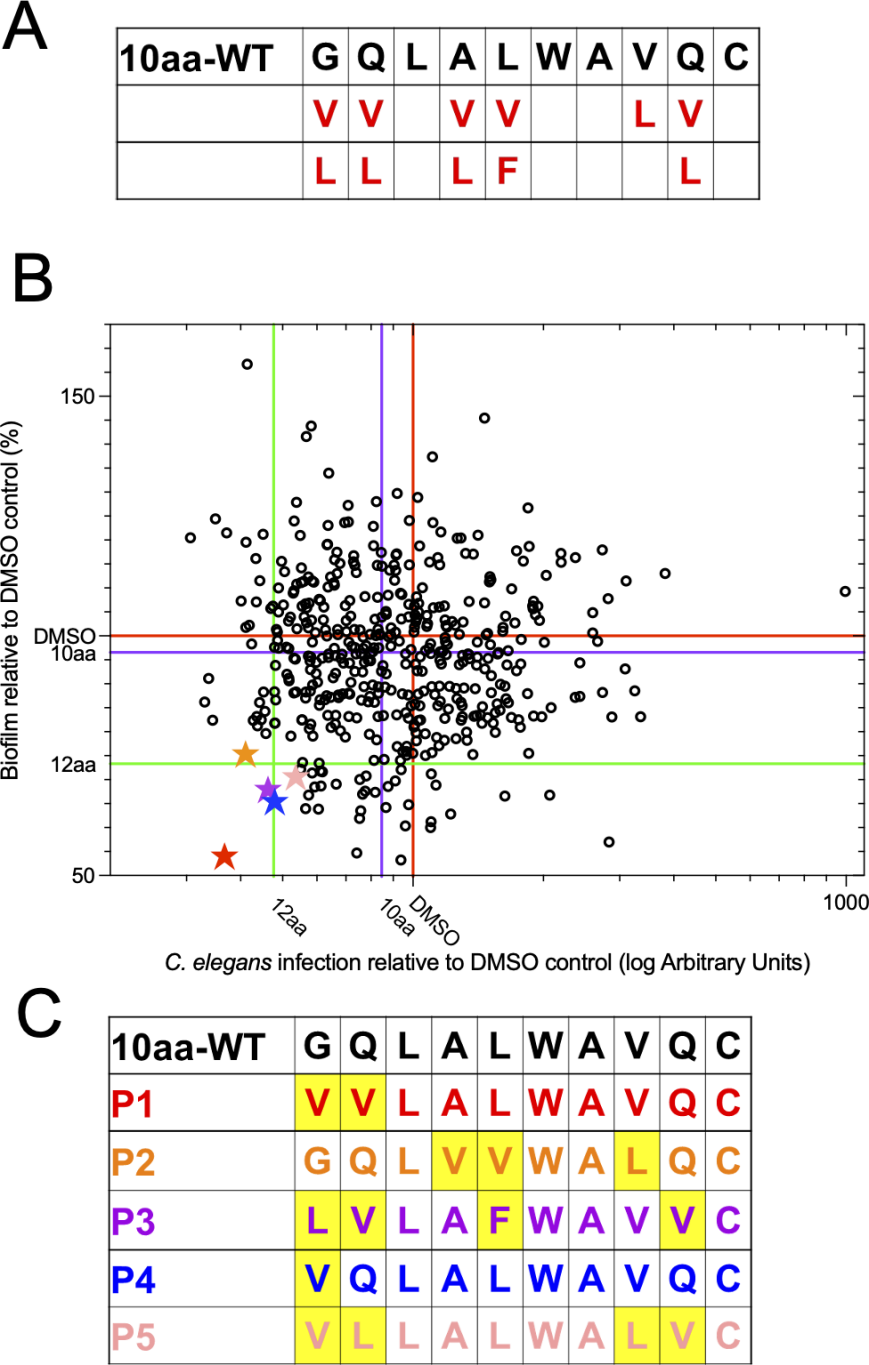
Combinatorial peptide library generation and high-throughput screening identifies five gain-of-function variants. (**A**) The synthesis map outlining the positions at which substitutions were made and the different amino acids added during combinatorial solid-phase peptide synthesis (SPPS) to generate the library of 486 new sequences. (**B**) A plot of the relative efficacy of the library-generated peptides in protecting *C. elegans* infected with *C. albicans,* as measured by the high-throughput SYTOX Orange assay (x-axis) and in reducing *C. albicans* biofilm formation (y-axis). Each peptide was tested in both experiments with three controls: the untreated DMSO control (red line), the 10aa parent sequence (purple line), and the 12-aa benchmark peptide for antifungal activity (green line). (**C**) Sequences of the five lead candidates, represented as stars in (**B**), compared to the parent 10aa wild-type sequence. The raw data used to generate this plot can be found in Table S2.

The results above suggest that a rational design by site-directed mutagenesis is unlikely to generate peptides with more activity. Based on the information gleaned from this mutational analysis, we decided to implement a combinatorial library screening approach to better understand the features of the peptide important for activity as well as to isolate more active variants. Relative to the fully functional 12aa segment of α7, the 11aa or 10aa peptides are less potent (29). However, these partially active variants present an opportunity to identify variants of shorter sequence having activity equal to or greater than that of the 12aa variant. This would be valuable as shorter sequences may be easier and less expensive to synthesize and have greater biofilm-penetrating capability. Thus, the 10aa variant was chosen as the initial parent sequence to generate a combinatorial peptide library as the first step in the process of synthetic molecular evolution, which was then screened for enhanced activity (30–34). Several positions along the native 10aa sequence were chosen as flexible positions to incorporate more hydrophobic residues (Fig. 2A); thereafter, split-and-recombine chemistry was carried out using solid-phase peptide synthesis to generate a library of 486 novel peptides (35–39).

Screening of this library was carried out by employing two established assays to assess the antifungal agent efficacy. The first was a high-throughput infection assay in which *C. elegans* was infected in 96-well plates, and the worm mortality is estimated after 5 days by the fluorescence intensity of the live/dead stain SYTOX Orange. Versions of this assay were previously used to screen compound libraries to identify novel antimicrobial agents (40–42) and to assess the virulence of *C. albicans* mutants (43). The second assay tested reduction in *C. albicans* biofilm formation (15). This screening approach allowed us to evaluate the peptide’s effects on two different aspects of *C. albicans* virulence (Fig. 2B). Each individual point on the graph represents a single peptide from the 10aa library, and the three colored lines on each axis represent the internal controls used to compare each library member: the 12aa peptide (green), the 10aa peptide (purple), and the DMSO control (red). The closer the peptide is to the bottom left portion of this sectioned plot, the better the observed antifungal activity of the individual library member. The raw data for this screening can be found in Table S2.

As expected, we found that most of the peptides from the library displayed activity equivalent to, or worse, than that of the 10aa parent peptide (Fig. 2B). However, three peptides named P1, P3, and P4 with “P” standing for positive hit, displayed equal or enhanced activity in both assays relative to the 12aa peptide, followed by peptides, P2 and P5, which were the next best in their performance, and hence additionally chosen for further analysis. The five peptides were sequenced by Edman degradation, and their divergence from the parent sequence is presented in Fig. 2C.

### Secondary confirmation of enhanced antifungal activity from the library hits

**Fig 3:**
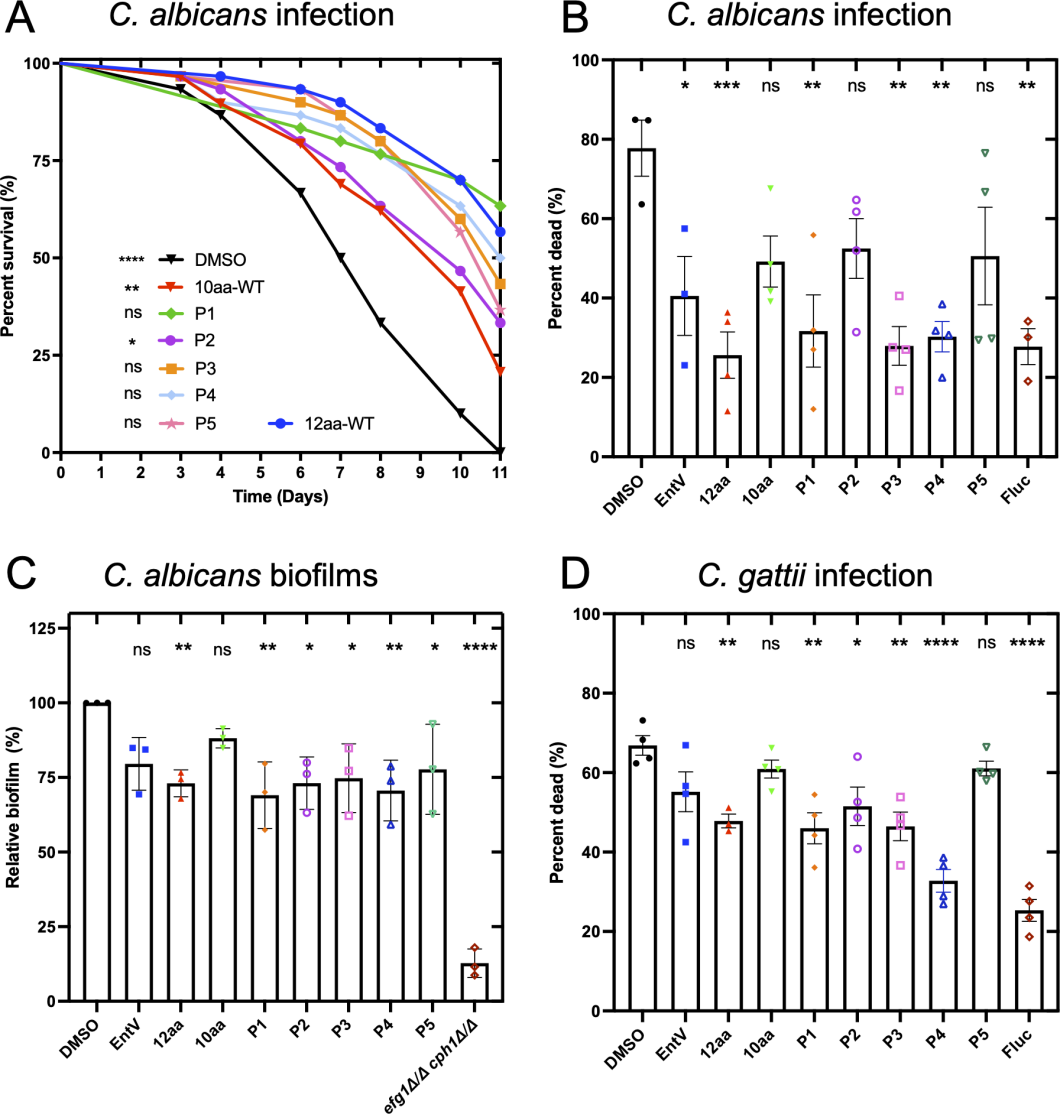
Secondary testing of gain-of-function variants from the screen. (**A**) Survival over time of *C. elegans* infected with *C. albicans* and exposed to 1 nM of the indicated peptides. Statistical differences in survival were compared to the animals treated with the 12aa-WTpeptide by Mantel–Cox log rank analysis. A total number of 30 animals were used, and one representative trial is shown. Median survival and *P* values of all trials are presented in Table S1. (**B**)The percent survival as measured by the high-throughput SYTOX Orange assay following 5 days of infection with *C. albicans* is shown. Means were calculated, with the error bars representing the SEM. Statistical differences in survival were compared to animals in the untreated condition (DMSO) by one-way ANOVA followed by Dunnett’s multiple comparisons test. One representative trial is shown with two biological replicates presented in Fig. S1. A total number of 30 animals were used per experimental group. (**C**) Biofilm formation in the artificial saliva medium following incubation with 10 nM of the indicated peptides is shown. A biofilm-deficient strain (*efg1∆∆cph1∆∆*) was used as a control. Means were calculated, with the error bars representing the SEM. Statistical significance in comparison to the DMSO control group was determined using one-way ANOVA followed by Tukey’s multiple comparison test. Three biological replicates, each with four technical replicates, were used. (**D**) Percent survival as measured by the high-throughput SYTOX Orange assay following 5 days of infection with 40–71 (*C. gattii*) is shown. One representative trial is shown with three biological replicates presented in Fig. S2. Thirty animals were used per experimental group. For all statistical tests, *P* values < 0.05 were considered statistically significant. *P < 0.05, **P < 0.01, ***P < 0.001, and ****P < 0.0001.

The synthesis of the combinatorial library using split-and-recombine chemistry resulted in only a small amount of each peptide being generated, enough for screening and Edman sequencing, but not enough for further testing. To validate the variant sequences, the five peptides were resynthesized and tested in the standard *C. elegans* survival assay after infection with *C. albicans*. Four of the five 10aa library hits (P1, P3, P4, and P5) were as effective in protecting the animals as the original 12aa peptide; the differences in survival were not statistically significant (Fig. 3A; Table S1). The SYTOX Orange assay was also employed. However, instead of using the fluorescence intensity metric that was used for high-throughput screening, we implemented an image analysis pipeline that allowed us to score individual worms and determine the percentage of dead nematodes. Compared to the untreated DMSO control, we found that three of the variants P1, P3, and P4 showed antifungal activity comparable to that of the 12aa peptide and a statistically significant improvement relative to the 10aa parent sequence (Fig. 3B; Fig. S1). Thus, the combinatorial library identified shorter peptides with enhanced activity.

The peptides were then tested for their ability to reduce biofilm biomass in the artificial saliva medium (15). Compared to the untreated DMSO control and in contrast to the 10aa parent variant, all five of the variant peptides demonstrated statistically significant antifungal activity (Fig. 3C). The variant peptides showed approximately a 20–30% decrease in biofilm biomass like the 12-aa peptide. The experiment also included the biofilm-deficient *efg1Δ/Δ cph1Δ/Δ* strain of SC5314 *C. albicans* as a negative control. These results demonstrate that the 10aa peptide variants have activity resembling that of the 12aa peptide, and significantly better than that of the original 10aa variant.

Finally, the variant peptides were tested in the SYTOX Orange assay against an evolutionarily divergent fungal species, *Cryptococcus gattii*, to assess the potential for broad-spectrum activity. While the 10aa parent sequence was not effective, we found that P1–P4 had significant antifungal activity when compared to the untreated DMSO control (Fig. 3D; Fig. S2). We additionally tested the best-performing 12aa variant, P4, against a reference *C. gattii* strain [R265 (44)] and the related species, *Cryptococcus neoformans* [H99 (45)], in the *C. elegans* survival assay and found that P4 was also effective against these strains (Fig. S3A). As EntV appears to inhibit the virulence properties of *C. albicans*, we investigated the impact of P4 on well-characterized virulence factors of Cryptococcal species, including growth at 37°C (Fig. S3B), capsule formation (Fig. S3C), and melaninization (Fig. S3D). No defects were observed, suggesting that the attenuation of virulence comes via another mechanism. In conclusion, the variant peptides identified from the library demonstrated broad-spectrum antifungal activity comparable to that of the 12aa parent variant, but in a manner that is neither fungistatic nor fungicidal, as previously observed for *Candida albicans* (15, 29).

### Antifungal efficacy of variant peptides in a murine model of oropharyngeal candidiasis (OPC)

**Fig 4:**
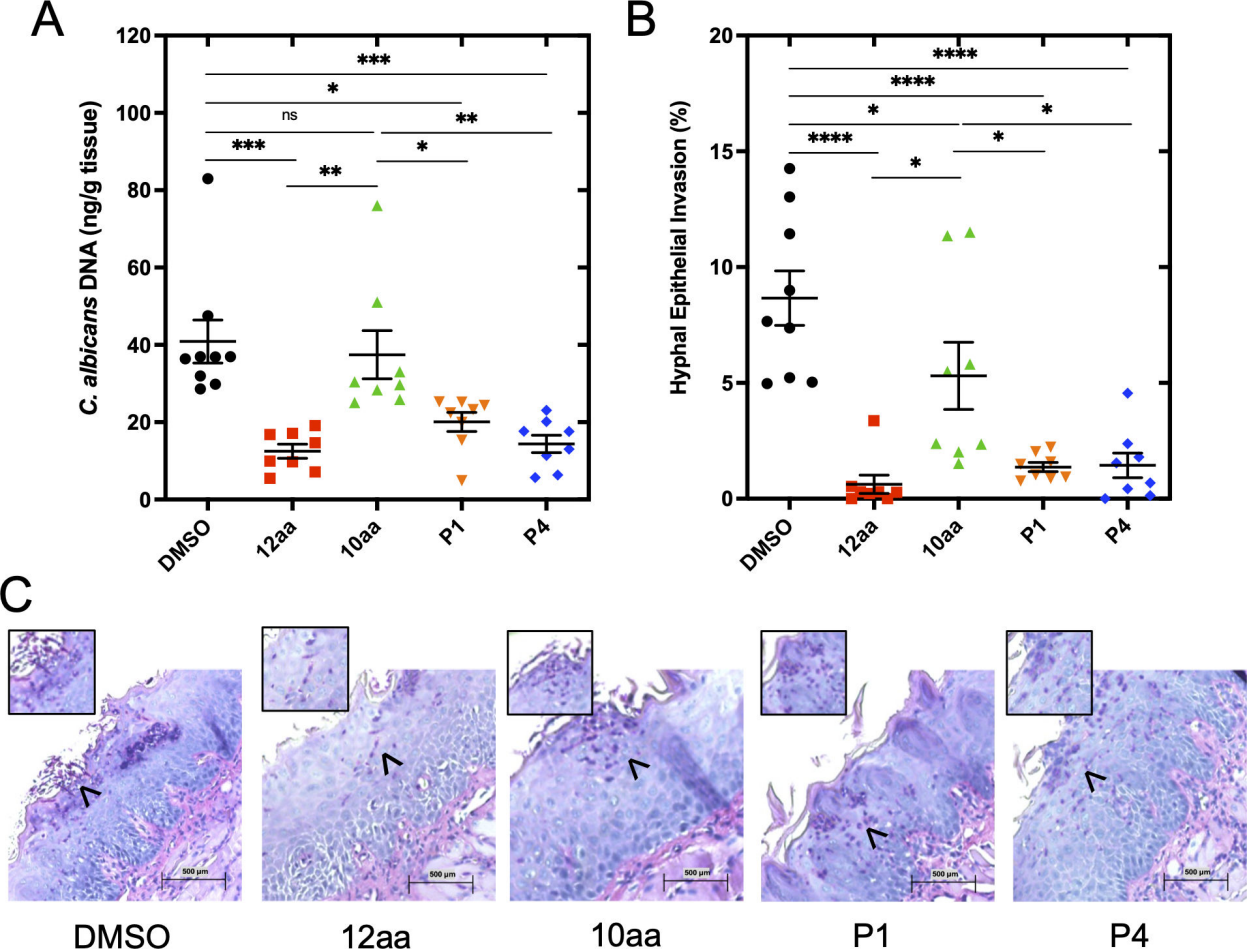
The 10aa variants P1 and P4 demonstrate enhanced protection in a mouse model of oropharyngeal candidiasis compared to the 10aa parent variant. The severity of OPC was determined by (**A**) the amount of *C. albicans* DNA detected by qPCR and (**B**) the percentage of the tongue surface showing hyphal invasion in animals given 100 nM of the indicated peptides or the vehicle control (DMSO) in their drinking water for 5 days following inoculation. Means were calculated, with the error bars representing the SEM. Survival differences were compared by one-way ANOVA followed by Tukey’s multiple comparisons test. For all statistical tests, *P* values < 0.05 were considered statistically significant. *P < 0.05, **P < 0.01, ***P < 0.001, and ****P < 0.0001. (**C**) Representative images used to score the hyphal invasion of the tongue tissue. *C. albicans* is represented by pink-stained cells in the areas that are magnified, indicated by the black arrows. The cells in the control DMSO sample show that the hyphal morphotype of *C. albicans* is more prevalent than the peptide-treated tissue samples.

Although all five peptides showed statistically significant antifungal activity in at least some assays, the P1 and P4 candidates were the best-performing in aggregate and were chosen for further testing. An established murine model of oropharyngeal candidiasis (see Materials and Methods) was used (15, 29, 46). Immediately following sub-lingual inoculation with *C. albicans*, the mice from each group were given water containing the peptides, which was consumed *ad libitum* for 5 days. After 5 days, the mice were euthanized, the tongues were excised, and the tissue was processed for fungal burden (via qPCR) and histological analyses. As shown in Fig. 4A and B, both the fungal burden and the percent of the epithelial surface showing hyphal invasion were significantly reduced when the P1 and P4 peptides were compared to the DMSO control and to the 10aa parent peptide (Fig. 4A and B). Representative histological images that were used to calculate percent hyphal invasion are shown in Fig. 4C. Arrows indicate *C. albicans* observed in purple against the background stain of the tongue tissue. A higher density of hyphal formation in the DMSO control and 10aa-treated mice was observed, whereas restriction to the yeast cell morphology was apparent in the mice treated with the other peptides. We note that there is some limited invasion into the epithelium of treated mice (Fig. 4C), but this is associated with neither hyphal growth nor neutrophil-mediated inflammation.

### Assessment of peptide stability and modifications for improvement

**Fig 5:**
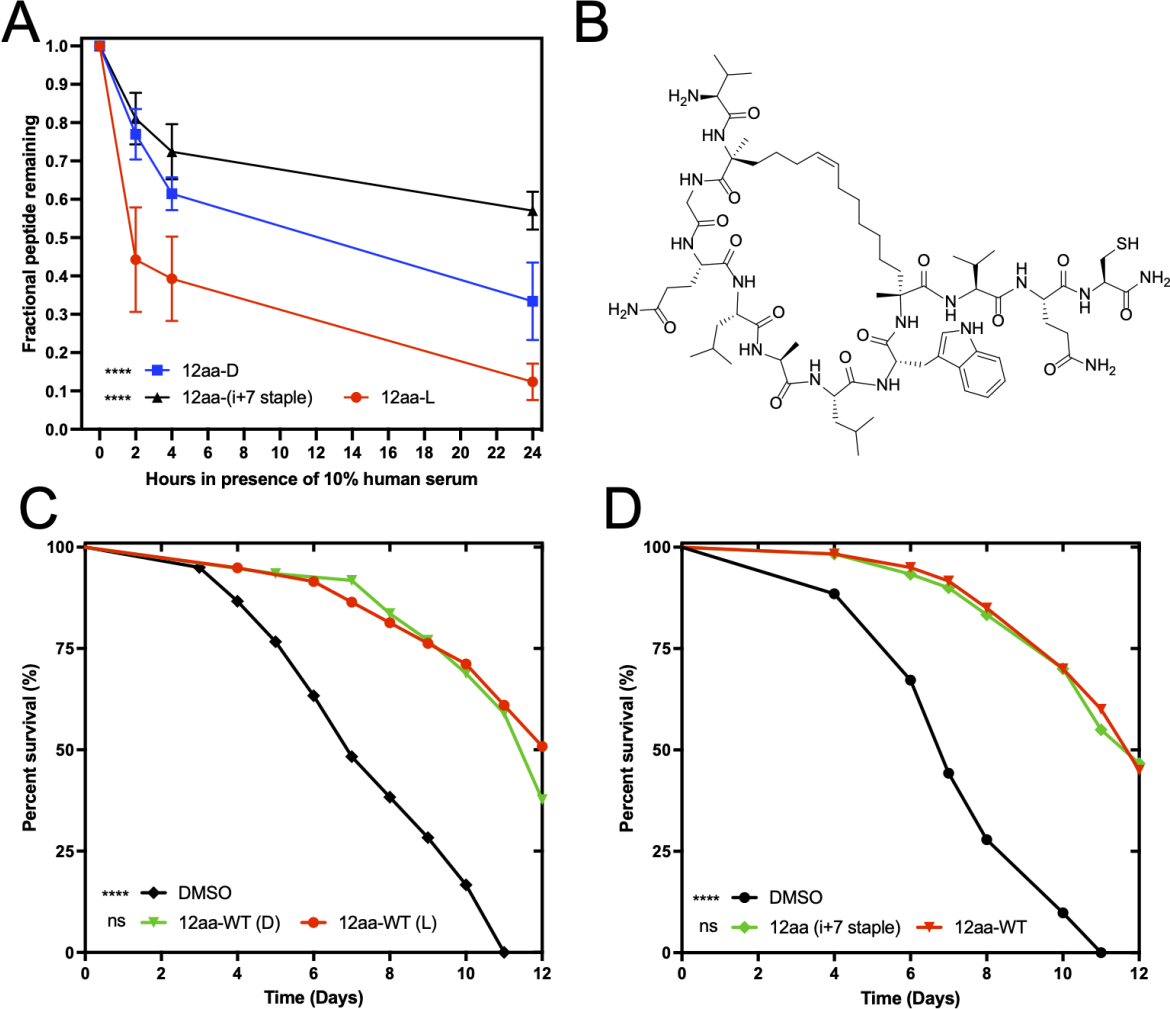
Modifications for increasing the stability of the 12aa peptide do not ablate antifungal activity. (**A**) Peptides were exposed to 10% human serum, and their stability over time was tracked using HPLC purification to measure the amount of the remaining peptide. A modified chi-squared curve analysis was used to compare the stability of the modified peptides to the native L-form 12aa peptide. The error bars represent the SEM. (**B**) The chemical structure of the I + 7 stapled peptide. (**C and D**) Survival over time of *C. elegans* infected with *C. albicans* and exposed to 1 nM of the indicated peptides. Methods and statistical analysis were the same as in Fig. 1, and extended data median survival and *P* values of all trails are presented in Table S1. For all statistical tests, *P* values < 0.05 were considered statistically significant. *P < 0.05, **P < 0.01, ***P < 0.001, and ****P < 0.0001.

We next aimed to test the peptide variants in a murine systemic model of candidiasis (29, 47, 48). However, a potential limitation of peptide antimicrobials is their lack of stability in physiological conditions, such as in the bloodstream where they are exposed to many proteases. Our previous work demonstrated that EntV was not particularly stable in 10% serum. It was quickly degraded by serum proteases, with only about 10% remaining after 4 hours of incubation (29). As shown in Fig. 5A, the 12aa variant was more stable, with 40% remaining after 4 hours, but only 10% remaining after 24 hours with an estimated half-life of 2.4 hours. Despite their relative instability in serum, EntV and the 12aa variant were still remarkably efficacious in protecting mice from infection when co-injected with *C. albicans* (29).

To test if stability could be further improved, several modifications were introduced into the 12aa variant. First, we generated the peptide with D-form amino acids, which are inherently more resistant to proteases and peptidases due to the nature of the peptide bonds (49–52). In fact, the 12aa-D peptide had notably improved peptide stability, with approximately 35% remaining after 24 hours in the presence of human serum and the estimated half-life improved to 14 hours (Fig. 5A). Next, we tested a variant of the 12aa peptide that uses a hydrocarbon staple to partially cyclize it. In short, we synthesized the peptide with modified alanine residues, taking advantage of the spacing of the naturally occurring alanines to introduce a covalent linkage between these side chains (53–55). The chemical structure of this peptide is shown in Fig. 5B. These types of hydrocarbon staples can only be used in short, helical peptides and must occur in the canonical i + 3, i + 4, or i + 7 positions to coincide with the number of residues in one or two turns of the helix (54). The purpose of this staple is twofold: it can lock the conformation of the secondary structure for the helical peptide, and it also sterically hinders the accessibility of the peptide backbone to proteases and peptidases (53–55). As shown in Fig. 5A, this modification was significantly stabilizing, resulting in approximately 60% of the original peptide being intact after the 24-hour incubation with an estimated half-life of 33 hours. Importantly, neither modification caused loss of antifungal activity. Both the 12aa D-form peptide and the stapled peptide remained as effective as the original in protecting *C. elegans* against infection with *C. albicans* (Fig. 5C and D; Table S1). In conclusion, modifications of the EntV truncated variants to improve resistance to proteases can be engineered.

### Antifungal efficacy of variant peptides in a murine model of systemic candidiasis

**Fig 6:**
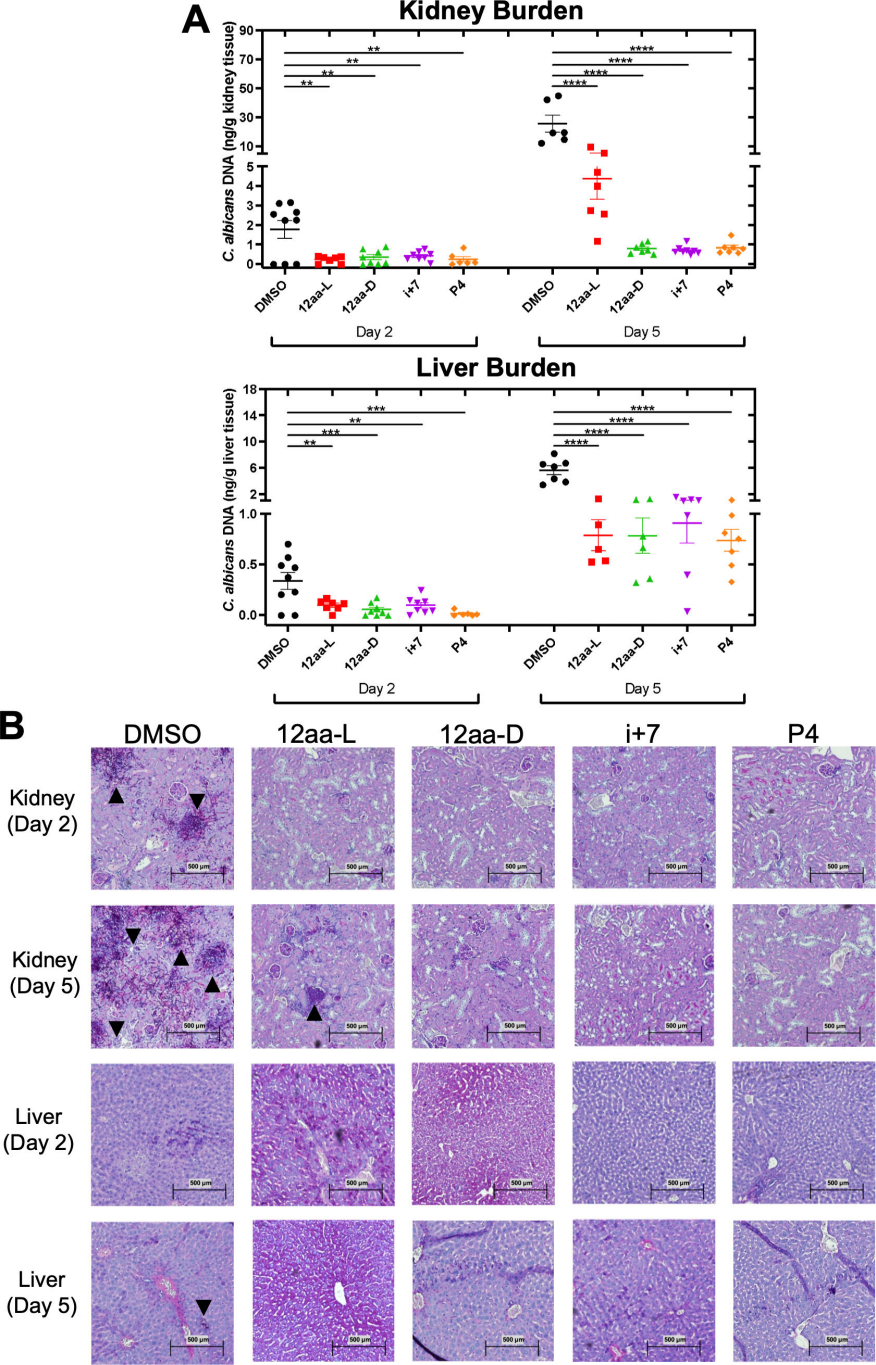
The stability-enhanced 12aa variants and the 10aa P4 variant have antifungal activity in a mouse model of systemic candidiasis. *C. albicans* was inoculated by tail vein injection following a 2-hour pre-incubation with 100 nM of the indicated peptides. (**A**) Fungal organ burden was assessed by measuring *C. albicans* DNA by qPCR in the kidneys (top) and liver (bottom). Means were calculated, with the error bars representing the SEM. Statistical differences were compared by one-way ANOVA followed by Tukey’s multiple comparison test for all samples, with only statistically significant differences indicated with lines. For all statistical tests, *P* values < 0.05 were considered statistically significant. *P < 0.05, **P < 0.01, ***P < 0.001, and ****P < 0.0001. (**B**) Representative images of the kidney and liver tissue, respectively, at Day 2 and Day 5. Foci of significant *C. albicans* growth are indicated by black arrowheads. Scale bars indicate 500 µM.

Next, these sequence and stability variants were tested in the murine systemic model of candidiasis, which mimics the most serious manifestation of human *Candida* infection (29, 47, 48). We chose the P4 10aa variant because it was more effective than P1 in reducing fungal burden in the OPC model (Fig. 4A). We also included the 12aa-D and 12aa (i + 7 staple) peptides, as they were more stable, while still efficacious (Fig. 5). The bloodstream candidiasis infection was established in outbred ICR mice with an intravenous inoculation through the lateral tail vein of 5 × 10^5^ CFUs of *C. albicans* pre-incubated with or without the peptide for 2 hours prior to the injection. Two points about this experimental design must be noted. First, pre-inoculation does not reduce the viable CFUs injected (29). Second, this experimental modality assesses the prophylactic potential of the peptide rather than the therapeutic potential (see *Discussion*). Endpoints of 2 days and 5 days post-infection were chosen to assess *C. albicans* burden in the kidney and liver of the mice, two organs that have particularly high fungal burdens in systemic infections. At the two endpoints, the mice were euthanized, and the right kidney and medial lobe of the liver were extracted from each mouse. The tissue was then bisected to be processed for qPCR and histological analyses. As shown in Fig. 6A, both on day 2 and on day 5, the fungal burden was significantly reduced in the kidneys for all peptide-pre-incubated inoculations compared to the DMSO (untreated) control. In addition to fungal burden, histological images were collected, and representative examples of hyphal formation in the kidneys are presented in Fig. 6B. Similarly, when assessing the liver burden, a statistically significant reduction was observed for all peptide-pre-incubated inoculations compared to the DMSO control (Fig. 6C); representative images are presented in Fig. 6D. Figure 6A and C have a segmented y-axis to observe the effects of the peptide more closely at the earlier day 2 timepoint, when the *C. albicans* burden was lower. The peptide treatments were also compared with one another and were not significantly different except for the day 5 kidney sample, in which the 12aa-L was significantly less effective compared to the other peptides. As indicated by the arrows in the histology panels in Fig. 6B and D, clusters of *C. albicans* with a high density of hyphal formation can be observed in the DMSO control, while less of *C. albicans* is observed in the treated samples. These data suggest that the variants with sequence and stability changes might be more effective than the 12aa parent peptide.

## DISCUSSION

The goal of this study was to further understand the features of EntV and EntV-derived α7 peptides necessary for activity in order to improve the efficacy and stability. Previous work had found that the cysteine is crucial and that the glutamines could be substituted for hydrophobic, but not positively charged, residues without loss of activity (27, 29). Here, we show by alanine scanning of the 12aa and 11aa variants that no additional individual amino acid side chain is crucial for activity. Based on these data, we hypothesized that changes that increased hydrophobicity might enhance activity. However, substituting all three alanines in the 12aa variant for valines or leucines reduced the activity (Fig. 1F), though we cannot distinguish whether this is from increased hydrophobicity or steric interference from the larger side chains. To further probe the structural requirements of this peptide and identify more active variants, we generated a combinatorial library in the weakly active 10aa variant. We made substitutions at six of the 10 residues (except for the crucial cysteine and the tryptophan used for fluorescent quantitation of the peptide) to replace the naturally occurring amino acid with a more hydrophobic alternative.

This library contained 486 peptides that were screened in the *C. elegans* and *in vitro* biofilm assays. Five hits were identified that demonstrated antifungal activity in our non-vertebrate assays to varying degrees. P1 and P4 had the most consistent increase in activity across all assays and so were further studied in vertebrate models. In the OPC model, both peptides reduced epithelial invasion and fungal burden in the tongue to a significantly greater degree than the 10aa parent sequence, and they were equivalent to the 12aa reference. In the murine systemic infection model, the 10aa P4 peptide showed protection comparable to or better than that of the 12aa original sequence, as measured by the kidney and liver burdens of *C. albicans*. Interestingly, both peptides had valine substitutions at the first glycine (position 1), with P1 having an additional valine substitution at the second residue, a glutamine. These data support the hypothesis that modest increases in hydrophobicity enhance the activity. Moreover, they indicate that positional changes in the N-terminal region of the short peptide may be most effective. Overall, these results confirm the ability of synthetic molecular evolution to select peptides with enhanced antifungal activity from parent sequences that may have moderate efficacy, such as the 10aa truncation of EntV, the parent sequence of the library.

The original 12aa peptide had limited stability in human serum, which may limit its bioactivity. We therefore generated chemical modifications that we predicted would reduce protease sensitivity, both a hydrocarbon staple (i + 7) and a peptide synthesized from the D-enantiomers (12aa-D). These modified peptides were moderately (12aa-D) or significantly (i + 7) more stable in serum, and both peptides were fully active in the biofilm and *C. elegans* assays. In the systemic model, mice given *C. albicans* incubated with the P4, 12aa-D, or i + 7 peptides all had markedly reduced fungal burdens in the kidney at days 2 and 5, relative to the untreated animals. At day 5, these modified peptides performed better than the native 12aa sequence, suggesting a possible increase in efficacy. The differences were less pronounced in the liver. Further testing of stabilized EntV variants for improved efficacy is an area of future interest. Additionally, it will be of interest to test a true treatment model where the peptide is given after systemic infection is established. We note that the 12aa peptide was effective in a treatment modality in the OPC model (29). Additional parameters of pharmacokinetics, pharmacodynamics, tissue penetration, and delivery routes, such as IV or intraperitoneal injections, also need to be investigated.

Though the focus was on *C. albicans*, we demonstrated that EntV and the variant peptides were effective in the nematode model against *C. auris* and *Crypotococcus* species here and in previous work (29). As *Cryptococcus* is a basidiomycete and thus separated from *Candida* by at least several hundred million years of evolution, this suggests a highly conserved target. Preliminary experiments from our laboratories indicate that EntV-derived peptides bind to the cell envelope of susceptible fungi to interfere in some way with the expression of virulence traits such as adhesion and biofilm formation. The sequence flexibility of the peptide suggests that the mechanism does not involve a “lock-and-key” inhibition of an enzymatic activity, but rather an interaction based more on hydrophobicity, perhaps binding to lipid or carbohydrate moieties on the cell surface. Understanding the mechanism of action of EntV and its strain and species range are high-priority areas of investigation.

In conclusion, this study highlights the possibility of optimizing known antifungal peptides beyond their native capabilities. Synthetic molecular evolution, combined with chemical modifications that improve peptide stability, can be used in concert to propel the development of clinically relevant antifungal peptides, an important goal considering the dearth of, and growing resistance to, currently available antifungals.

## MATERIALS AND METHODS

### Strains and media

Fungal strains were propagated in BHI & YPD media. Most experiments used *C. albicans* wild-type strain SC5314 (56). For the biofilm assays, the non-adherent strain HLC54 (*efg1∆∆cph1∆∆*) was used as a negative control (57). *Cryptococcus* strains utilized include 40–71 (a gift of L. Ostrosky), R265 (44), and H99 (45).

### Solid-phase peptide synthesis and combinatorial peptide library generation

Unless otherwise indicated, each peptide used in the study was synthesized by solid-phase peptide synthesis using established methods in FMOC amino acid additions (36, 39). Briefly, starting from the C-terminal residue, the peptides were synthesized on TentaGel S-Ram 0.2–0.8 meq/g beads (from Chem Impex). The combinatorial peptide libraries were synthesized on TentaGel NH2 macrobeads (300 µm particle size with 0.23 mmol/g capacity) using split-and-pool methodologies in combinatorial chemistry, described in detail elsewhere (30, 33, 35, 37, 38). FMOC-protected peptide monomers (Advanced ChemTech) were dissolved in dimethylformamide (DMF) at roughly a 2.5 x molar excess (0.528  mM) relative to the manufacturer’s stated loading capacity (0.22  mM). The reactions were catalyzed by the addition of hexafluorophosphate benzotriazole tetramethyl uronium (HBTU) (200.11  mg per residue addition), hydroxybenzotriazole (HOBt) (71.28  mg per residue addition), and N, N-diisopropylethylamine (DIPEA) (68  µL per residue addition). Reaction completion for each residue was confirmed by conducting a ninhydrin test on a small sample of beads taken from the reaction vessel after washing the beads three times with DMF to clean any residual or unreacted components. Following completion of amino acid addition, the beads underwent a final deprotection to remove the FMOC group and then were washed with dichloromethane (DCM). Acid cleavage from the beads was accomplished by using Reagent B (88% v/v trifluoroacetic acid, 5% v/v phenol, 5% v/v ddH2O, and 2% v/v triisopropylsilane). The validation of synthetic procedures was done using HPLC, mass spectrometry, and Edman degradation sequencing. The library peptides were beta-branched to increase the capacity and then synthesized using a photocleavable linker attaching the peptide to the bead, which could be cleaved with exposure to UV light for 2 hours on dry beads in a single layer in a glass Petri dish. The beads released a concentration of roughly 9 µM peptide in 50 µL of DMSO.

### *C. elegans* infection assays

*C. elegans* infection assays were performed with strain *glp-4(bn2);sek-1(km-4)* (40), which were propagated and maintained on *E. coli* strain OP50 grown as lawns on nematode growth medium (NGM) agar using standard techniques (58). To synchronize the nematodes to the same stage of growth, L1 stage worms on non-starved plates were washed off, filtered through a 10- µm filter (pluriSelect, pluriStrainer 10  µm), harvested by centrifugation at 1,500 rpm for 60  seconds, transferred to OP50-seeded plates, and grown to the L4 stage.

To prepare the infection plates, fungal strains were grown in BHI broth overnight at 30°C with agitation. About 500 µL of the culture was plated onto BHI solid medium containing gentamycin (10 µg/mL) and grown for 24  hrs at 37°C. The synchronized L4 *C. elegans* nematodes were then washed off the OP50 plates in 2  mL sterile M9 buffer and washed once, centrifuging at 800 rpm for 30  seconds to collect the nematodes. Nematodes were infected by placing them on the fungal lawn for 4  hrs at 25°C. Following this exposure, they were washed off the plate and washed four times with 2  mL of sterile M9. The nematodes were then pipetted (~30 per well with two wells per condition for a total of ~60 worms assayed) into six-well plates with 2  mL of the liquid medium (20% BHI broth and 80% M9) containing 1 nM of the peptide. A DMSO-untreated control was used with each experiment. Plates were incubated at 25°C, and worm death was scored daily. Kaplan–Meier survival curves were generated and analyzed, as described in the quantification and statistical analysis section.

A modified version of the *C. elegans* survival assay described previously using live/dead staining was also employed (41, 42). The preparation of the L1 stage nematodes to the infection stage was the same. However, post-infection, the animals were placed into 96-well plates with roughly 50 animals per well and incubated at 25°C for 5 days in the absence or presence of the 1 nM peptide. Following this incubation, they were washed off the plate and washed four times with 200  µL of sterile M9 and placed into a black 96-well plate with clear bottoms.

At this stage, SYTOX Orange (a membrane-impermeant DNA-intercalating dye) was prepared at a concentration of 3  µM in an S basal buffer, and the worms were stained with the dye overnight. Fluorescence and brightfield images were taken of each well using a BioTek Cytation 5 Imaging Reader, and two metrics were used in the analysis of this assay: infection ratio (arbitrary units) and percent dead (%). The infection ratio was calculated as fluorescence units/brightfield units = infection ratio. This metric was used for high-throughput screening of the peptide library by approximating the peptide efficacy via nematode death signaling by SYTOX Orange. Post-screening, the metric of percent dead (%) was used to get more precise measurements. This was done by counting the number of worms that were illuminated on the SYTOX Orange channel (dead worms) and dividing them by the number of worms observed in the brightfield channel (total worms).

### Biofilm assays

The methods used for these experiments are outlined elsewhere (15, 59). Briefly, *C. albicans* strains were grown in YPD broth for 18  hrs at 30°C with agitation and then sub-cultured for 4  hrs in the same conditions. Cells were then collected by centrifugation at 16,000 x g for 30  s, washed twice in PBS, and adjusted to a concentration of 5 ×  10^6^ cells/mL (measured with Countess II, Life Technologies) in PBS treated with the 10 nM peptide. The cell suspension was incubated for 1  hr at 30°C with agitation at 200 rpm. Then, 100  µL of the cell suspension was added to wells of a 96-well tissue culture-treated polystyrene plate (Falcon). The PBS and unadhered cells were removed and then replaced with 200  µL of the artificial saliva medium (15, 60) containing the 10 nM peptide. The plate was then incubated at 37°C for 48 h.

After incubation, the medium was gently removed along with any planktonic cells. The biofilm was then stained for 20  min with 50  µL of 0.08% crystal violet solution (Sigma). The crystal violet stain was then aspirated, and the wells were washed four times with sterile water. Biofilms were de-stained with 200  µL of 200 proof ethanol for 20  min, and 100  µL of the ethanol solution was transferred to a new well for analysis. The optical density at 595  nm (OD_595_) was measured using a Synergy H1 plate reader (BioTek Cytation 5) with Gen5 version 3.08 software (BioTek).

### *Cryptococcus* capsule and melaninization measurements

For capsule measurements, *Cryptococcus* strains were grown in Saboraud broth and incubated at 30°C with shaking overnight. The cells were centrifuged at 870 x g for 5 min and washed twice with phosphate-buffered saline (PBS). The capsule was induced by incubating 5 × 10^6^ cells/mL in 5 mL 1/10 Saboraud +50 mM MOPS (pH 7.3) at 30°C shaking (200 rpm) for 48 h. Post-incubation, the cells were centrifuged again, and all but a small amount of the supernatant was aspirated. The pellet was resuspended in the remaining supernatant, and 1 µL was added to 0.4 µL of India ink. Following gentle mixing, the cells were applied to a microscope slide, and images were obtained at 100 x. Using the Count and Measure function in cellSens software (Olympus), the thickness of the capsule for each cell was measured by drawing three vectors between the cell wall and the end of the capsule and averaging. A total of 30 cells/condition were measured, and the Mann–Whitney test (Prism 9.0) was used to determine if there were significant differences between capsule thickness distributions.

To assess melanin production, *Cryptococcus* cells were grown in YPD overnight at 30°C shaking. They were then diluted to 2 × 10^7^ cells/mL in PBS and incubated with and without the peptides for 2 h at 30°C shaking. To induce melaninization on the solid medium, strains were plated on chemically defined minimal medium (15 mM dextrose, 10 mM MgSO_4_, 29.4 mM KH_2_PO_4_, 13 mM glycine, and 3 µM thiamine, pH 5.5) or Saboraud dextrose agar (2% dextrose and 1% peptone) with and without 1 mM L-DOPA (D9628 Sigma–Aldrich, St. Louis, MO, USA). Serial 1:10 dilutions were performed, and 5 µL of each was spotted on plates and incubated at 30°C, protected from light, and visually assessed for melanin production at the indicated time points (Fig. S3D).

### Murine oropharyngeal candidiasis model

The efficacy of 12-mer and 10-mer variant peptides was tested in the OPC model (15, 29, 46). BALB/c mice, 10 weeks old and roughly 18–20 g in weight, were immunosuppressed by injecting 225  mg/kg cortisone acetate subcutaneously 1 day before inoculation and subsequently on days 1 and 3 post-inoculation. To prepare the inoculum, 1  mL of the *C. albicans* (SC5314) overnight culture grown at 30°C in YPD broth was washed twice in PBS before resuspension in sterile Hanks’ Balanced Salt Solution (HBSS) at a concentration of 1  ×  10^6^ cells/mL. Calcium alginate swabs were soaked in this inoculum for 5  min prior to inoculation. Mice were anesthetized using ketamine and xylazine and placed on pre-warmed isothermal pads, and the swabs were placed sublingually for 75  min. Mice were given additional doses of ketamine (50  mg/kg), as necessary. After inoculation, mice were given drinking water with the 12-mer and 10-mer variant peptides or DMSO (0.01%) *ad libitum*. Mice were euthanized on the fifth day after inoculation. The tongues were then excised and cut in half laterally for tissue histology and assessment of fungal burden. All animal procedures were conducted in accordance with the protocols approved by the Animal Welfare Committee of the University of Texas Health Science Center at Houston.

For tissue histology, half the tissue sample was placed in 10% zinc-buffered formalin overnight and stored in 80% ethanol, before embedding in paraffin. For each tissue, 5-µm sections were prepared using a Leica microtome and stained using Periodic Acid-Schiff (PAS) stain. All sections were scanned at 40 x magnification for histopathological analysis using a light microscope. The other half of the tongue was homogenized for assessment of fungal burden via qPCR. DNA was extracted by using the Yeast DNA Extraction Kit (Thermo Scientific), and qPCR was used for amplifying a 269-bp fragment of the internal transcribed spacer 2 (ITS2) between the 5.8S and 28S ribosomal RNA genes of *C. albicans*. The qPCR was performed with the FastStart Universal SYBR Green master mix with the ROX kit (Roche) using a CFX96 Real-Time System with a C1000 Touch thermal cycler (BioRad). To screen for contamination and background fluorescence during qPCR amplification, no template controls were used.

### Murine systemic candidiasis infection model

Based on our previous methodology (29, 47, 48), the systemic candidiasis infection model was applied by growing *C. albicans* SC5314 overnight in YPD media at 30°C, then diluting this culture media at a ratio of 1:100, and growing it again in a second overnight culture. The late-log phase culture was collected by centrifugation at 16,000 x g for 30  s, washed in PBS, and diluted to 1  ×  10^7^ cells/mL in PBS containing the vehicle (0.01% DMSO) or 100  nM peptide and incubated for 2  hrs at room temperature. Female ICR mice, 8 weeks old and roughly 18–20  g in weight, were used. Mice were inoculated via the lateral tail vein with a 50-µL injection. Animals were monitored at least twice daily for signs of moribundity and euthanized if necessary (any euthanized animals were excluded from subsequent analysis). At days 2 and 5, the mice were sacrificedeuthanized, and organs were harvested for assessment of fungal burden. The right-side kidney was collected from each mouse as was the median lobe of the liver. Histology and qPCR were each conducted on half of the tissue sample, as outlined previously in the OPC method section. All animal procedures were conducted in accordance with protocols approved by the Animal Welfare Committee of the University of Texas Health Science Center at Houston.

### Quantification and statistical analysis

GraphPad Prism 9.0 was used for all data analysies. For the fungal adhesion assay, means of the experimental conditions were calculated and compared to the DMSO condition. Lines with error bars indicate the mean and the standard error of the mean (SEM). Significance was determined using one-way ANOVA followed by Dunnett’s multiple comparisons test. For the OPC model and systemic infection model fungal burden and % hyphal invasion measurements, means of the experimental conditions were calculated and compared to other conditions, as indicated in the individual panels. Lines with error bars indicate the mean and the standard error of the mean (SEM). Significance was determined using one-way ANOVA followed by Tukey’s multiple comparisons test. The median survival and comparison values for all *C. elegans* survival experiments and their replicates can be found in Table S1. For comparisons of the *Cryptococcus* capsule thickness with and without the peptide, the two-tailed *P* value was calculated using the Mann-–Whitney test. For all statistical tests, *P* values < 0.05 were considered statistically significant, and asterisks in the figure panels indicate the levels of significance as follows: *P  <  0.05, **P  <  0.01, ***P  <  0.001, and ****P  <  0.0001.

## Data Availability

No large data sets were generated that require deposition into public repositories. All data are in the manuscript with the source data underlying the figures in Table S2.

## SUPPLEMENTAL MATERIAL

Supplemental figures mbio.00570-24-s0001.pdf Figures S1-S3.

Table S1 mbio.00570-24-s0002.xlsx Statistical analysis and additional biological replicates of *C. elegans* survival curves.

Table S2 mbio.00570-24-s0003.xlsx Source data underlying figures.

ASM does not own the copyrights to Supplemental Material that may be linked to, or accessed through, an article. The authors have granted ASM a non-exclusive, world-wide license to publish the Supplemental Material files. Please contact the corresponding author directly for reuse.
