# Supplementary material for: Optimization of the antifungal properties of the bacterial peptide EntV by variant analysis
Source: mBio. 2024 Apr 9;15(5):e00570-24. doi: 10.1128/mbio.00570-24 (PMC11077972; doi:10.1128/mbio.00570-24)
Supplement: Supplemental figures — Figures S1-S3. [file mbio.00570-24-s0001.pdf]

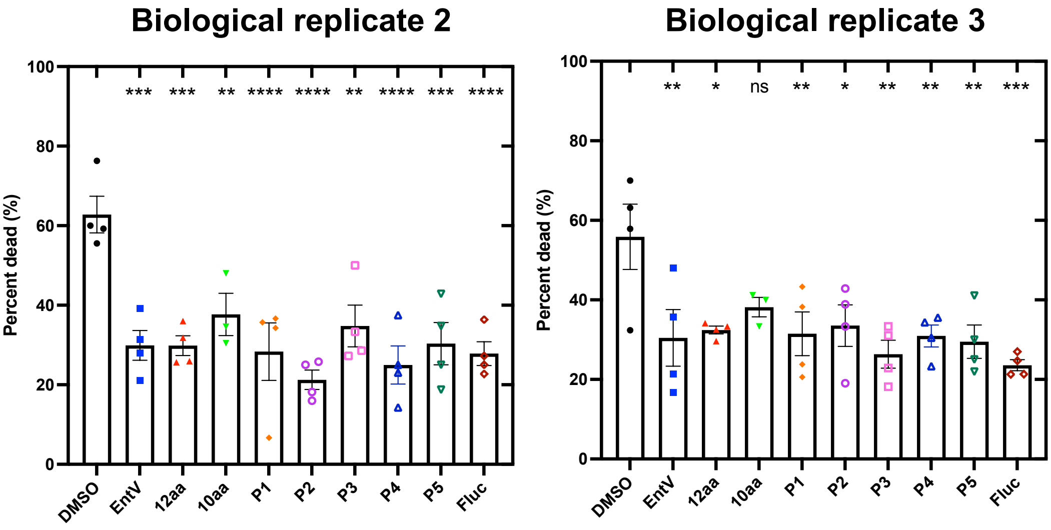

Fig. S1: Additional biological replicates of the *C. albicans* infection assay with *C. elegans* using Sytox Orange. Two additional biological replicates of the experiment presented in Fig. 3B were performed to assess percent survival as measured by the high-throughput Sytox Orange assay following five days of infection with *C. albicans* and the indicated treatments. Methods and statistical analysis were as described in Fig. 3. For all statistical tests, p values < 0.05 were considered statistically significant. *$p < 0.05$, **$p < 0.01$, ***$p < 0.001$, ****$p < 0.0001$.

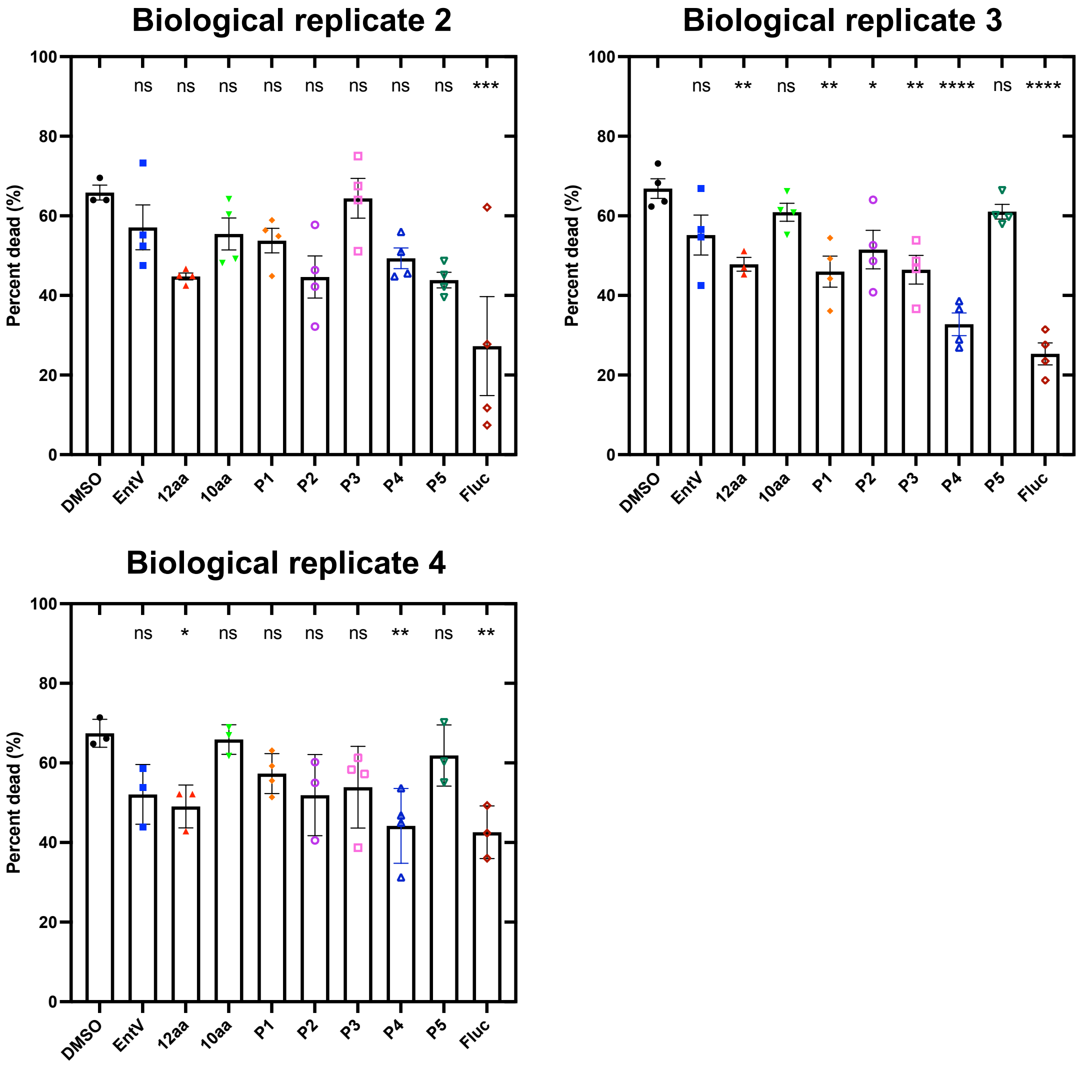

Fig. S2: Additional biological replicates of the *C. gattii* infection assay with *C. elegans* using Sytox Orange. Three additional biological replicates of the experiment presented in Fig. 3D were performed to assess percent survival as measured by the high-throughput Sytox Orange assay following five days of infection with *C. gattii* and the indicated treatments. Methods and analysis were the same as in Fig. 3. For all statistical tests, p values < 0.05 were considered statistically significant. *$p < 0.05$, **$p < 0.01$, ***$p < 0.001$, ****$p < 0.0001$.

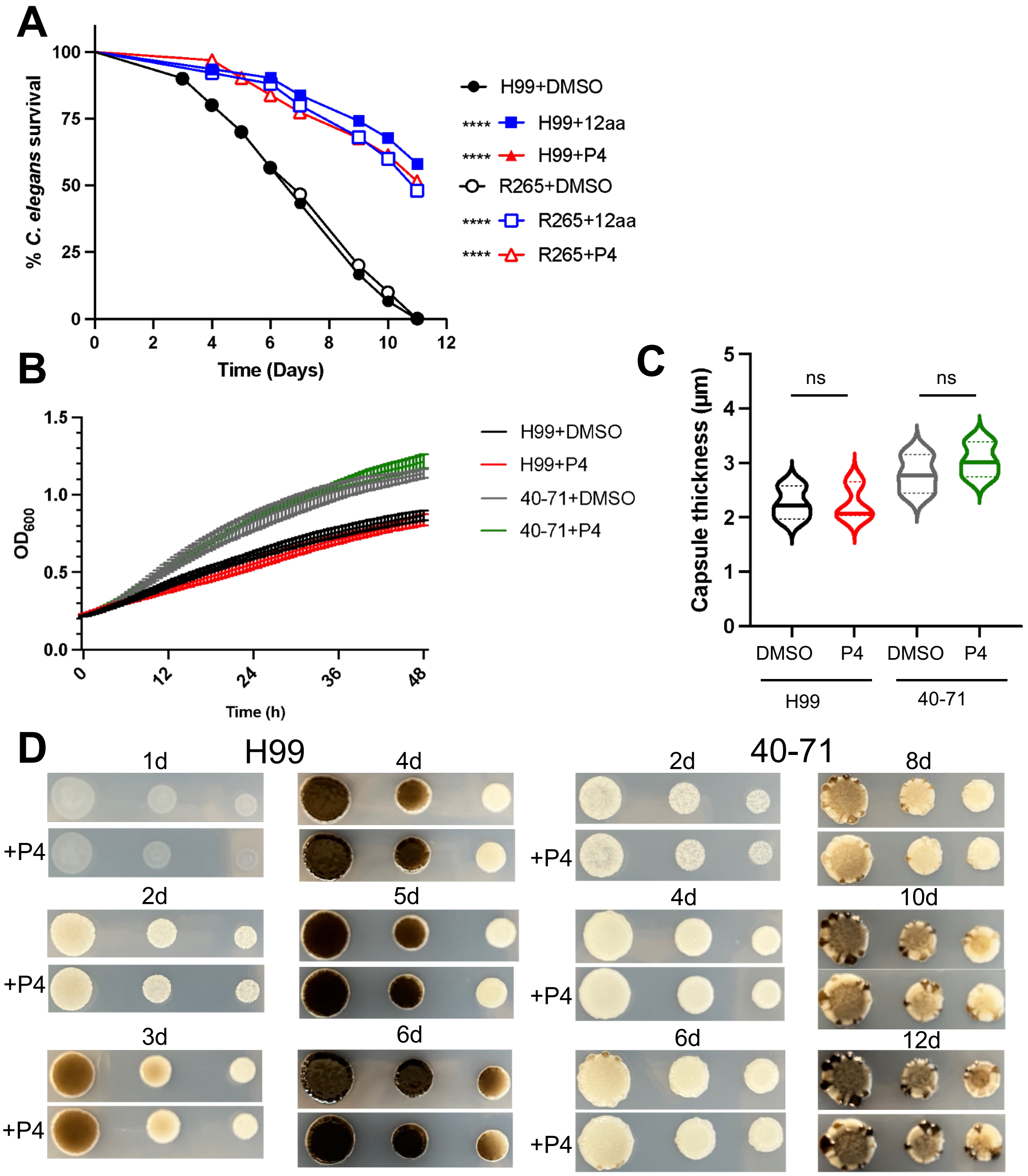

Fig. S3: P4 protects *C. elegans* from *Cryptococcus* infection without affecting growth, capsule formation, or melaninization. (A) Survival over time of *C. elegans* infected with strain H99 (*C. neoformans*) and R265 (*C. gattii)* and exposed to 1 nM of the indicated peptides. Statistical differences in survival were compared to the animals treated with the vehicle control (DMSO) by Mantel-Cox log rank analysis. An n of 30 animals was used and one representative trial is shown. Median survival and *p* values of all trials are presented in Table S1. (B) OD_{600} measurements of growth over time of H99 and 40-71 (*C. gattii)* in YPD with and without 1µM P4 grown at 37°C. (C) Capsule thickness medians and distributions for H99 and 40-71 following exposure to P4. A two-tailed Mann-Whitney test was used to compare the capsule thickness distribution to that of the unexposed controls. An n of 30 cells was used. (D) Representative pictures of melaninization of H99 and 40-71 exposed to 100nM P4 compared to control conditions. For all statistical tests, *p* values < 0.05 were considered statistically significant. **p* < 0.05, ***p* < 0.01, ****p* < 0.001, *****p* < 0.0001.
